# Developing a Portable Fluorescence Imaging Device for Fish Freshness Detection

**DOI:** 10.3390/s24051401

**Published:** 2024-02-22

**Authors:** Zheng Pan, Min Huang, Qibing Zhu, Xin Zhao

**Affiliations:** Key Laboratory of Advanced Process Control for Light Industry, Ministry of Education, Jiangnan University, Wuxi 214122, China; 6211905034@stu.jiangnan.edu.cn (Z.P.); zhuqib@163.com (Q.Z.); xinzhao@jiangnan.edu.cn (X.Z.)

**Keywords:** fish freshness detection, fluorescence imaging, FPGA, YOLOv4-tiny, low-cost, rapid

## Abstract

Rapid detection of fish freshness is of vital importance to ensuring the safety of aquatic product consumption. Currently, the widely used optical detecting methods of fish freshness are faced with multiple challenges, including low detecting efficiency, high cost, large size and low integration of detecting equipment. This research aims to address these issues by developing a low-cost portable fluorescence imaging device for rapid fish freshness detection. The developed device employs ultraviolet-light-emitting diode (UV-LED) lamp beads (365 nm, 10 W) as excitation light sources, and a low-cost field programmable gate array (FPGA) board (model: ZYNQ XC7Z020) as the master control unit. The fluorescence images captured by a complementary metal oxide semiconductor (CMOS) camera are processed by the YOLOv4-Tiny model embedded in FPGA to obtain the ultimate results of fish freshness. The circuit for the YOLOv4-Tiny model is optimized to make full use of FPGA resources and to increase computing efficiency. The performance of the device is evaluated by using grass carp fillets as the research object. The average accuracy of freshness detection reaches up to 97.10%. Moreover, the detection time of below 1 s per sample and the overall power consumption of 47.1 W (including 42.4 W light source power consumption) indicate that the device has good real-time performance and low power consumption. The research provides a potential tool for fish freshness evaluation in a low-cost and rapid manner.

## 1. Introduction

Fish products are widely consumed all over the world due to their richness in nutrients, such as proteins, vitamins, and unsaturated fatty acids [1,2]. As a typical fish product, fish fillets are increasingly consumed as a key ingredient in ready-to-cook dishes, driven by improving living standards and a fast-paced lifestyle [3]. However, fish fillets with high moisture content and abundant nutrients are susceptible to deterioration caused by endogenous and microbiological enzymes [4]. Breaks in the cold chain caused by unregulated practices still occur frequently throughout the cold chain transportation, resulting in spoilage of fish fillets [5,6]. Therefore, rapid and effective techniques for freshness detection of fish fillets are significant in preventing food safety risks.

To overcome the shortcomings of traditional methods (e.g., microbiological and chemical analytical methods) [7], various freshness detection technologies have been introduced, such as biosensors [8], electronic noses [9], electronic tongues [10], colorimetric sensor arrays [11,12], and optical sensing [13]. Over the years, optical sensing has become the most attractive measurement technique for fish freshness evaluation due to its simple sample preparation, noninvasiveness and fast measurement. Regarding fish freshness detection, the main optical sensing techniques include spectroscopy [14] and hyperspectral imaging (HSI) technology [15]. Spectroscopy technology, such as near-infrared (NIR) spectroscopy [16], fluorescence spectroscopy [17] and Raman spectroscopy [18], only collect information around a small area of the measured sample, which can introduce freshness measurement errors if there is a positional deviation between the measurement and spoilage areas. HSI technology, integrating the advantages of spectroscopy with machine vision, can simultaneously obtain the spatial and spectral information of the measured samples and overcome the weakness of spectroscopy. HSI technology has been widely reported in freshness evaluation of all types of meat, including fish [19,20]. However, the HSI systems used in existing studies generally suffer from the disadvantages of expensive equipment, large system size, and poor real-time detection due to large amounts of data acquisition and processing. These shortcomings bring difficulties to the wide application of HSI in meat freshness detection. Therefore, the development of low-cost, portable imaging equipment for rapid meat freshness testing remains an urgent task.

Two tasks need to be addressed in the development of a low-cost portable imaging device for rapid detection of meat freshness. One is developing a low-cost hardware system for rapid acquisition and process of optical image information reflecting meat freshness, and the other is building a freshness evaluation model embedded within the hardware system, which is used to reflect a mapping relationship between the acquired optical information and the freshness level of the sample. For the first task, it has been shown extensively that the freshness of meat can be characterized by a small number of images with wavelengths distributed in the visible/near-infrared spectral region, multispectral imaging devices for meat freshness detection can be developed based on these small number of wavelength images to increase the speed of detection and to reduce the price of the devices. However, the spectral cameras used in multispectral imaging devices are still expensive compared to RGB cameras used in conventional machine vision. Meat spoilage is accompanied by the production and accumulation of fluorescent substances such as Schiff bases, nicotinamide adenine dinucleotide, and porphyrin [21]. Fluorescence sensing can measure concentrations down to one-thousandth of what can be measured by normal absorption spectroscopy and thus has higher detection sensitivity [22]. Recently, Wu et al. [23] found that high-precision prediction of total volatile basic nitrogen (TVB-N) can be achieved with images of a small number of wavebands that are distributed in three spectral regions, namely, red, green, and blue (RGB). This feature facilitates the acquisition of RGB images using ordinary complementary metal oxide semiconductor (CMOS) cameras without the need for expensive spectral cameras. However, to the best of the authors’ knowledge, no research has been reported on the development of meat freshness detection equipment using low-cost CMOS cameras in combination with fluorescence technology.

For the second task, most of the meat freshness evaluation models constructed based on optical information are traditional machine learning algorithms, such as partial least squares regression (PLSR) and support vector machine (SVM). For example, Zhuang et al. [24] used the PLSR to construct a prediction model for fluorescence hyperspectral images and pork freshness indicators and reported a correlation coefficient of 0.92 and 0.88 for TVB-N and TVB-C, respectively. These model constructions mainly rely on manual experience to extract features from optical images, leading to the accuracy of subsequent evaluation models being influenced by the adequacy and validity of feature generation. Recently, some studies using deep neural networks for fish detection have been reported [25,26], which automatically extract features through deep learning to maximize the potential features of the data, resulting in significant improvements in detection accuracy and robustness, but these models are complex and usually need to be installed on computational resources with advanced performance.

In this study, inspired by the work of Wu et al. [23] and developments in deep learning, a low-cost portable fluorescence imaging device for fish freshness detection is developed based on a field-programmable gate arrays (FPGA) board. The developed device utilizes a low-cost CMOS camera and an FPGA master unit to achieve effective acquisition of fluorescence images of fish samples and deploys a deep learning model (namely, YOLOv4-Tiny) for fish freshness detection by exploiting the advantages of FPGA, such as parallel executions of multi-tasks, the reusability of hardware, and reallocation of memory and computing resources [27,28,29]. The developed system helps to promote the on-the-ground application of optical sensing technology in the meat industry. The specific objectives are:

- To develop a low-cost fluorescence imaging device for fish freshness detection based on FPGA hardware;
- To construct a fish freshness detection model based on a deep neural network called the YOLOv4-Tiny model and complete its circuit design and optimization;
- To evaluate the performance of the developed device by using grass carp fillets.

## 2. Materials and Methods

### 2.1. Fluorescence Image Acquisition Device

**Figure 1:**
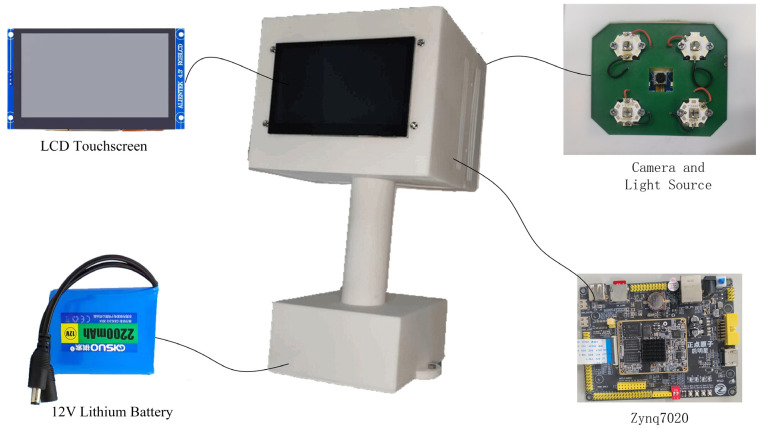
Fluorescence image acquisition device.

The developed fluorescence image acquisition device is shown in Figure 1. The main components of the device include a Zynq7020 development board (Guangzhou Star Wing Electronic Technology Co., Ltd., Guangzhou, China), an excitation light source, an image acquisition module, a power supply module, a memory module, and a display module.

The core chip of the Zynq7020 development board is ZYNQ XC7Z020, it consists of a processing system (PS) and a programmable logic (PL). The PS side includes a dual armCortex-A9 with a maximum operating frequency of 766 MHz, and dual data rate 3 synchronous dynamic random access memory (DDR3 SDRAM). It is mainly used for the control of the algorithm inference process and the display of the resulting image. The PL side is used to design the hardware circuit, which is mainly used to collect the fluorescence image data of fish and realize the hardware acceleration of the algorithm circuit of fish freshness detection. The high-speed Advanced Extensible Interface (AXI) bus is used for high-speed data transfer interaction on the PS side and the PL side. Inspired by the study of Lee et al. [30], four UV-LED lamp beads with a central wavelength of 365 nm and a nominal power of 10 W (Shenzhen Yindin Technology Co., Ltd., Shenzhen, China) are selected as excitation source to ensure that fluorescent substances in fish were fully excited. The image acquisition of the camera module uses a CMOS camera (model: OV5640) (Guangzhou Xingyi Electronic Technology Co., Ltd., Guangzhou, China), and the size of the captured image is set to 640 × 480 pixels. A 12V lithium battery powers the device. A 16 Gb SD card is used to store network parameters and fish freshness test results. The display module uses a 4.3-inch LCD touch display with an output resolution of 800 × 480. The function of image display and human–computer interaction is realized by ARM cross-compiling Qt. These components are all housed in portable housing.

**Figure 2:**
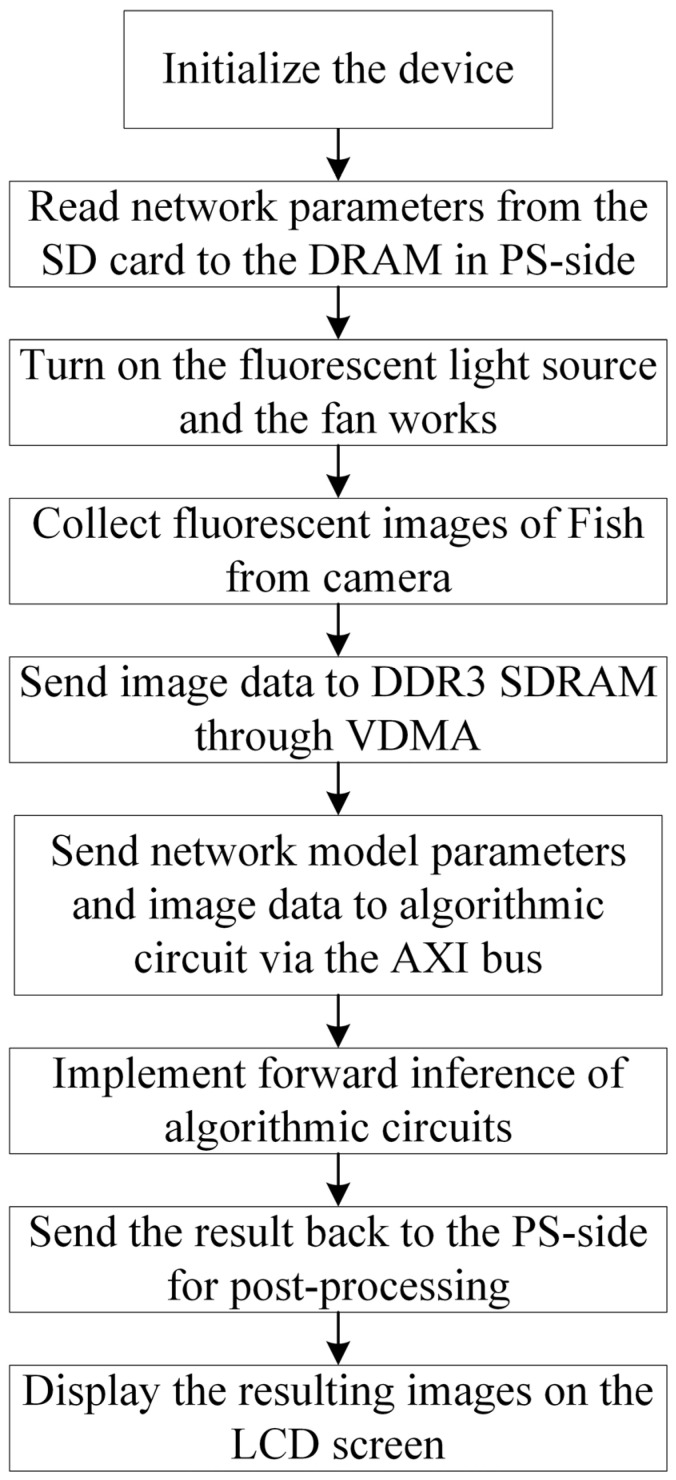
Flowchart of fish fluorescence image freshness detection.

Figure 2 summarizes the device operation process for fish freshness detection. After system initialization, the PS side reads the network model parameters from the SD card to the DRAM. Subsequently, the fluorescent light source is turned on, and the fan works. Next, the PL-side circuit starts the camera to acquire fluorescence images, and the acquired image data are stored in the DDR3 memory. Then, the stored network model parameters and image data are read into the algorithmic circuit via the AXI bus, and the PS-side code controls the PL-side algorithmic circuit to realize the algorithmic forward inference. Finally, the result is sent back to the PS side for image processing, and the resultant image is displayed on the LCD screen.

### 2.2. YOLOv4-Tiny Model

**Figure 3:**
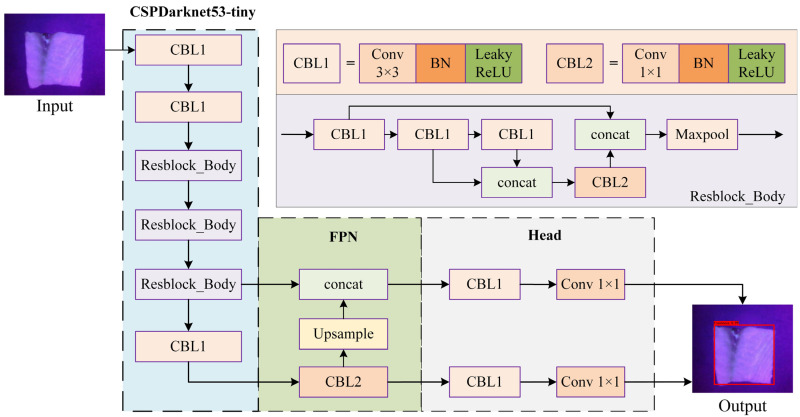
Structure of YOLOv4-Tiny model.

In addition to the developing hardware of the device, it is equally important to construct a real-time and accurate freshness detection model. To deploy deep neural network models on embedded devices for fast and accurate detection of fish freshness, the deep neural network model should have low complexity, and its computation and storage resources can be implemented through a general-purpose FPGA. The YOLOv4-Tiny model is a lightweight version of YOLOv4, with slightly reduced accuracy; but the model size is only 1/10 of the original, and the detection speed is significantly improved, which is fully suitable for rapid detection on embedded platforms with low computing resources. Based on this consideration, the YOLOv4-Tiny lightweight network shown in Figure 3 is selected [31].

The YOLOv4-Tiny model consists of a backbone network of CSPDarknet53-tiny for image feature extraction, a feature pyramid network (FPN) for multi-scale feature fusion, and a head network for classification. The CSPDarknet53-tiny is made up of three ‘CBL’ modules and three ‘Resblock_Body’ modules in series. The CBL structure is a module composed of a convolution layer, normalization processing and an activation function. The activation function uses the Leaky-ReLU function, simplifying the model parameters and increasing the calculation speed. The Resblock_Body structure is equivalent to the combination of residual networks and CSP. By dividing the input features into two parts, one part is retained, and the other part enters the residual network for processing, because only one residual structure is stacked, simplifying the model size and the number of parameters. After that, the results of the residual part and the retained part are spliced to obtain more feature information. The FPN performs feature fusion on the outputs of the last two modules of the CSPDarknet53-tiny network to ensure that the network obtains more feature details and rich semantic information. Finally, the head network obtains recognition results based on the output features of the FPN.

### 2.3. Circuit Design of YOLOv4-Tiny Model

The circuit of YOLOv4-Tiny is designed and coded using high-level synthesis (HLS) language [32]. HLS uses a language similar to C/C++ to program FPGAs, thereby increasing the level of abstraction and greatly reducing the time required for FPGA development. The YOLOv4-Tiny model is mainly composed of 3 × 3 convolution, 1 × 1 convolution, upsampling, downsampling (2 × 2 MaxPooling), and concat operations. The above calculations are divided into two categories. The first category, including 3 × 3 convolution and 1 × 1 convolution, accounts for more than 95% of the total computation volume of the network. The second category is some lightweight operations, such as upsampling, downsampling, and concat operations.

**Figure 4:**
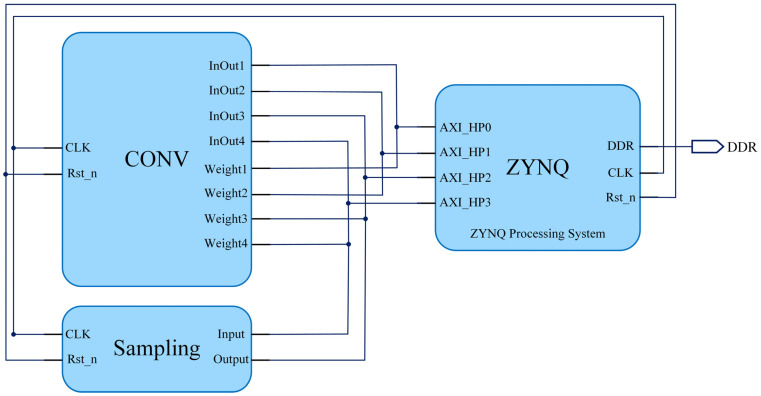
YOLOv4-Tiny circuit structural design.

In the HLS, the circuit code is encapsulated into two IP cores, the CONV IP core, and the sampling IP core, according to the above categorization. These IP cores are interconnected with the ZYNQ processing system in Vivado 2018.3 through four AXI HP (high-speed) interfaces. Data can be transmitted on the read-and-write data buses simultaneously between the memory from the PL and PS sides. The YOLOv4-Tiny circuit structural design is shown in Figure 4.

In YOLOv4-Tiny, the operation of each type of computation is the same; only the input or output size of each layer is different. Therefore, the reusable idea is adopted in the design of the circuit of the YOLOv4-Tiny. The CONV IP and sampling IP cores are instantiated. The PS side flexibly invokes the IP cores of the PL side according to the network structure sequence to realize the forward reasoning of YOLOv4-Tiny.

The CONV IP design is the core of algorithmic circuit design. The convolution process includes loading input and weight data, convolution operation, and storage and export of output data. The convolution circuit is divided into the memory access part and the calculation part according to the above process.

(a) Memory access part: All the data to be processed are stored in the external DDR3 SDRAM. Data are cached in an on-chip buffer before being provided to the processing unit due to limited on-chip resources. However, when accessing image data, FPGA block RAM (BRAM) cannot cache the entire feature map, so the block strategy is adopted. The input feature block of a specific size is calculated each time, and the output feature block is obtained by convolution. After the calculation is completed, the next input feature and weight are read and calculated. At the same time, the calculated output feature blocks are stored in the DDR3 SDRAM.

(b) Calculation part: Convolutional calculation is a multiplication and addition operation between input and weight data. This part of the circuit can be designed to take advantage of the parallel operation of FPGAs. One approach involves the implementation of parallel input and output channels. This method allows for the simultaneous calculation of convolutions involving inputs and weights across multiple input channels, as well as the results or partial sums of multiple output feature maps. The other approach is to parallelize the convolution window, parallelizing the multiplication between K × K neurons (K is the size of the convolution kernel) and the weights in the convolution window, and designing the process of the convolution operation as a parallel multiplication unit plus an additive tree structure, which fully exploits the parallelism of FPGAs.

### 2.4. Circuit Optimization Strategies

In the calculation operation and data reading and writing strategy of the algorithmic circuit, a lot of for-loop code is used. However, for-loop code is a serial structure and does not take advantage of the parallelism of an FPGA. Optimization of the algorithmic circuitry is required to reduce the calculation amount of the code and improve the parallelism of the code [33]. This study used the for-loop optimization strategy to optimize the cyclic data processing and calculation operations in the algorithm in parallel. Meanwhile, the strategies of fixed-point quantization, array partition, and ping-pong operation are also used to optimize the circuit.

#### 2.4.1. For-Loop Optimization

**Figure 5:**
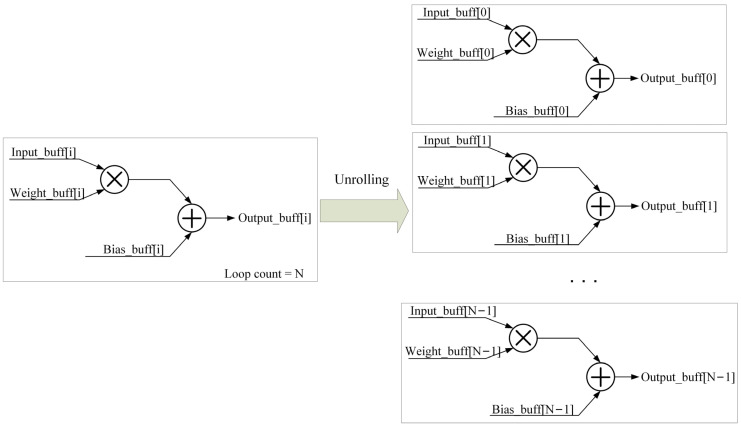
For-loop unrolling.

For-loop optimization operations are mainly used to increase the parallelism of the hardware system by unrolling and pipelining loops. Taking multiplication and addition operations in convolution as examples, the principle of unrolling the for-loop is shown in Figure 5.

**Figure 6:**
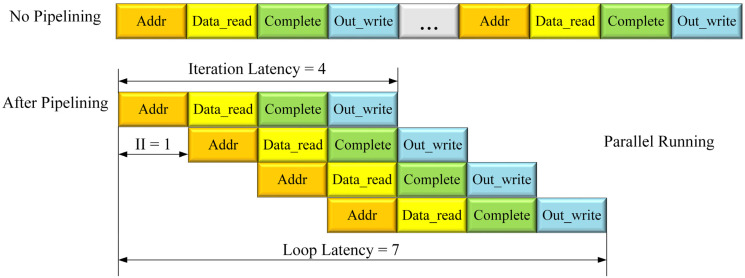
For-loop pipelining.

The for-loop codes are synthesized to generate the appropriate functional circuits, which by default use the same circuits for each loop. For-loop unrolling is copying the circuit into N copies, allowing all iterations to occur in parallel. However, this strategy takes up more resource space and is not suitable in all cases. When the for-loop optimization involves the reading and writing of data, optimizing the for-loop pipeline enables the FPGA to process a large amount of data synchronously as much as possible. The for-loop pipeline principle is shown in Figure 6.

The operation processes of different loop iterations are overlapped to improve the throughput of the system. When the for-loop pipeline is not used, the entire operation of reading data address, reading array data, calculating, and writing the output data are performed in order. After adopting the for-loop pipeline, the iteration latency is still 4, but the cycle initiation interval is reduced to 1, and the corresponding loop latency is reduced from 16 (4 × 4, iteration latency × for-loop count) to 7. A new input is processed each clock cycle to achieve the effect of parallel running, greatly reducing the loop latency.

#### 2.4.2. Other Optimization Strategies

Fixed-point quantization can reduce the amount of computation and the use of computing resources. In FPGA, the synthesized multiplier takes up the resources of the DSP. The YOLOv4-Tiny weight data are represented as 32-bit floating-point numbers, which need three DSP resources to complete multiplication and addition operations. However, the DSP unit is more adept at handling fixed-point arithmetic. Fixed-point operation requires only 1 DSP to realize multiplication and addition operations, which spend fewer resources, run faster, and cause negligible loss of neural network accuracy.

Array partition splits data into segments, and stores them on multiple small RAMs to improve reading and writing efficiency. The data of input and output feature blocks and intermediate parameters are temporarily stored in FPGA on-chip storage resources, but the data read and write ports of RAM or FIFO memory are limited. When multiple circuits read multiple data from one BRAM, parallel data access will cause conflicts. In this study, the parallelism dimension of the input–output feature block arrays and weight arrays is completely partitioned by the array partition strategy, which splits a large BRAM into multiple smaller RAMs. Data access conflicts are eliminated by reading data from each small RAM, which can effectively increase the number of storage read and write ports and improve data throughput.

Ping-pong operation is performed in reading and writing data to further increase the system throughput rate. The on-chip buffer is partitioned into two sections during the input and output stages of feature maps. Buffer 0 is used for computing the current feature block, whereas buffer 1 is used for reading or writing the feature block data. These buffers are alternately deployed to mask the data transmission time.

## 3. Experimental Methods and Data Acquisition

### 3.1. Experimental Sample and Image Acquisition

Taking grass carp fillets as the experimental object, fresh grass carp were purchased from China Resources Vanguard Supermarket in Wuxi, China. The fish bodies were bled and slaughtered, removing the scales and viscera, and were transported to the laboratory within 30 min. After quickly cleaning the blood stains on the grass carp with deionized water, the head and skin were removed. Then, the fish was cut into two pieces from the backbone, and the fishbones were removed at the belly. The fish was cut into 3 mm fillets of equal thickness using an automatic fish slicer machine (Hebei Xingtai Zhuozhen Machinery Factory, Hebei, China), and the fillets were then cut into 60 samples of the same size (40 mm × 40 mm × 3 mm, length × width × height). 

**Figure 7:**
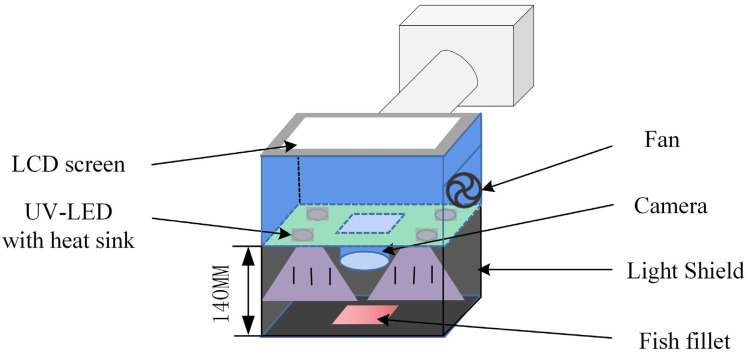
Schematic of fluorescence image acquisition device.

As seen in Figure 7, the blackboard carrying the experimental samples (grass carp fillets) was placed inside a black shade with a height of 140 mm to decrease the interference from external light sources. The fluorescence imaging device was placed on the top of the shade, and images of the front and back of the grass carp fillets were captured separately. The experiment was conducted for a total of 8 days. A total of 120 fluorescence images (60 × 2) were collected on day 0 (freshly slaughtered fillets). After image collection, 5 samples were randomly selected for standard value determination, and the remaining samples (55 fish fillets) were sealed using a self-sealing bag and placed in a refrigerator at 4 °C. On the second day, all samples were taken out from the refrigerator, fluorescence images of all fish fillets were collected, and five samples were randomly selected for standard value determination. The above procedures were repeated for 8 days, resulting in 565 fluorescence images.

### 3.2. Measurement of TVB-N Values

**Figure 8:**
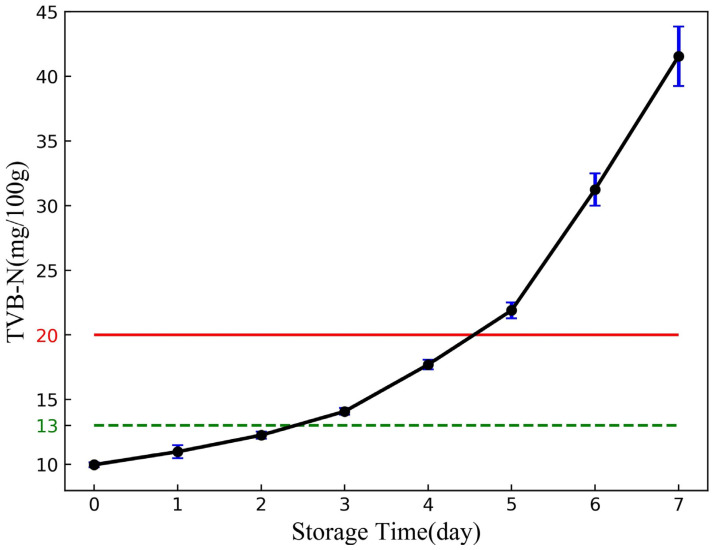
Change curve of the average TVB-N value of grass carp fillets in 8 days.

For each day, five samples were used for TVB-N measurement by the semi-micro Kjeldahl method (GB 5009.228–2016, China National Standard) [22,34], and some procedures were slightly modified. Five grams of fish samples were homogenized in a homogenizer with 50 mL of distilled water for two minutes. The homogenized mixture was poured into a digestion tube and left to stand for 30 min, then 1 g of magnesium oxide powder was added to the digestion tube, and the content of TVB-N in grass carp fillets was determined by K9840 automatic nitrogen analyzer (Hainan Future Technology Group Co., Ltd., Hainan, China). The curve of TVB-N content including mean value, maximum and minimum value for grass carp fillets stored at 4 °C is given in Figure 8.

According to Figure 8, there was a slow increase in TVB-N content on days 0–2, but the values were less than 13 mg/100 g, and then there was a large increase in TVB-N content, and after day 5, all the TVB-N content exceeded 20 mg/100 g. According to the Chinese National Standard for Food Safety (GB 2733-2005), the freshness grade of freshwater fish can be classified into three grades according to the TVB-N content: freshness (TVB-N value ≤ 13 mg/100 g), secondary freshness (13 mg/100 g < TVB-N value ≤ 20 mg/100 g), and rot (TVB-N value > 20 mg/100 g). Thus, for the experimental samples in this study, fish fillets in 0–2 days, 3–4 days and 5–7 days can be defined as freshness, secondary freshness and rot, respectively. According to the above strategy, a total of 565 samples include 193 fresh samples, 173 secondary fresh samples and 199 rotten samples. All of the samples were divided into training samples and test samples in a ratio of 3:1, resulting in 424 training samples for model training and 141 test samples for model performance evaluation.

### 3.3. Evaluation of Model Performance

To fully reflect the performance of the detection algorithm. Select “Precision”, “Recall”, “F1 Score” and “Average Precision” to evaluate the model’s detection performance. Precision (P) defines the ratio of fish images with correct freshness detected to the total number of fish images with correct and incorrect freshness detected. Recall (R) defines the ratio of fish images with correctly detected correct freshness to the total number of fish images with correctly detected and undetected correct freshness. The F1 score is the weighted average of precision and recall. Their formulas are expressed as follows:

(1) $$P=\frac{TP}{TP+FP}\times 100\%$$

(2) $$R=\frac{TP}{TP+FN}\times 100\%$$

(3) $$F1=\frac{2\times P\times R}{P+R}\times 100\%$$

where TP is the number of positive samples that correctly identify the corresponding freshness, FP is the number of samples that predict the correct freshness of negative samples, and FN is the number of samples that predict the non-corresponding freshness of positive samples. The precision and recall are based on the threshold value of 0.5.

The Average Precision (AP) is the average value of precision at different recall rates, which is equal to the area under the precision–recall curve, and is expressed as:

(4) $$AP=\int_{0}^{1}P(R)dR$$

## 4. Results and Discussion

### 4.1. Fluorescence Image Analysis

**Figure 9:**
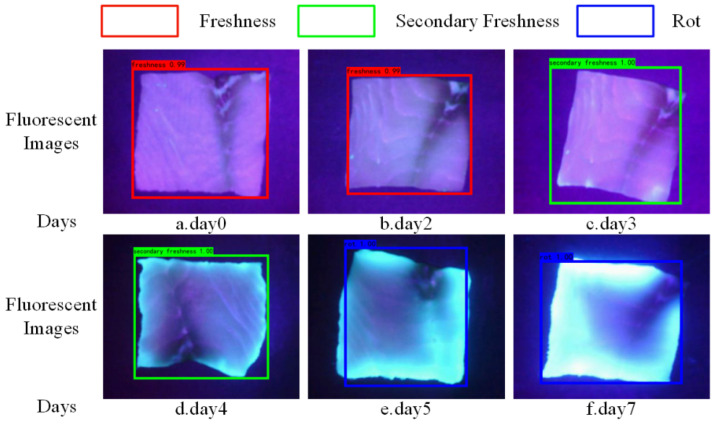
Fluorescence image detection of grass carp fillets.

Figure 9 shows the changes in fluorescent images and the corresponding freshness detections of grass carp fillets over time. The red box indicates that the sample of grass carp fillet is fresh, the green box indicates that the sample is secondary fresh, and the blue box indicates that the sample is rotten.

As can be seen from Figure 9, the freshness grade of grass carp fillets decreases with time. The surface of the fish fillet gradually produces blue fluorescence, and the fluorescence region expands from the edge to the center and becomes more prominent and brighter. This trend may be related to microorganisms and the fluorescent substances produced by metabolic processes. Pseudomonas is one of the most important bacteria in meat spoilage. Pseudomonas fluorescens is the main cause of food spoilage and emits blue fluorescence under UV-LED irradiation [35].

### 4.2. Fish Freshness Detection Results Using Developed Device

The model performance is shown in Table 1, the average accuracy of the YOLOv4-Tiny in detecting the freshness of grass carp fillets is high, and it can correctly identify grass carp fillets with different freshness levels. Good performance indicators were achieved when the classes were freshness and rot, with the APs of 99.71% and 97.89%, respectively. When the class is secondary freshness, the AP is slightly lower, but it is also above 93%. The mean AP (mAP) of the model reaches 97.10%.

**Figure 10:**
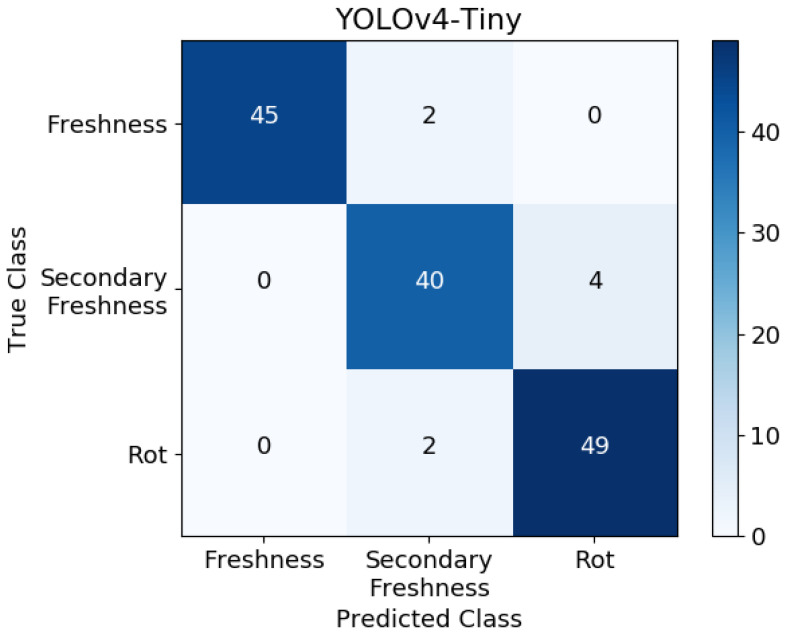
YOLOv4-Tiny confusion matrix for fish freshness detection.

The confusion matrix in Figure 10 shows the classification results of YOLOv4-Tiny on the test samples for different freshness levels of grass carp fillets. The misclassifications are mainly concentrated on adjacent grades.

**Figure 11:**
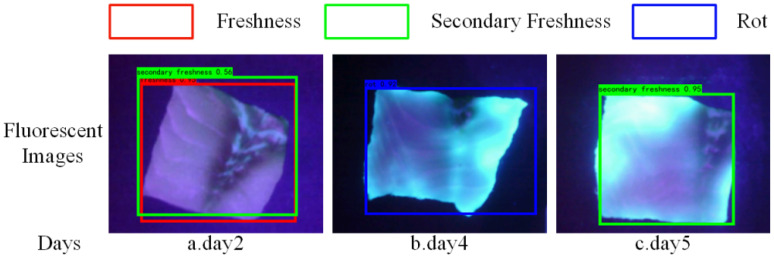
Fluorescence image samples of grass carp fillet freshness misclassification.

Figure 11 shows examples of misclassification by YOLOv4-Tiny, the top is the prediction boxes and the bottom is the actual storage days of the sample. Specifically, (1) in Figure 11a, which shows a fluorescence image of a fresh fish fillet on day 2, the probability of the fillet being detected as fresh is 0.95, and the probability of it being detected as secondary fresh is 0.56. Although the probability of detection as fresh is much higher than that of secondary fresh, there are two prediction boxes shown because both are higher than the prediction threshold of 0.5. In Figure 11a, there are white banded areas on the surface of the fillet left after removing the skin of the fish. Due to the small number of similar samples, YOLOv4-Tiny may misidentify these features resulting in misclassification. (2) Figure 11b shows a fluorescence image of a second fresh fillet erroneously detected as a rotten fillet on day 4, where the fish fillet has already shown a clear fluorescence reaction. The cause of the misclassification may be related to the long exposure of the sample to air during the experiment, which accelerated the spoilage of the fish and led to a mismatch between the labeled freshness of the fillet and its true freshness, resulting in an error. (3) Figure 11c shows a fluorescence image of a rotten fillet on day 5 that was erroneously detected as a secondary fresh fillet, although there is a clear fluorescence response on the image, the right side of the fillet is the area described in Figure 11a above leading to a weaker fluorescence response on the right side of the fillet. YOLOv4-Tiny recognizes not only features such as fluorescence intensity but possibly also features such as fluorescence area, leading to misclassification.

In summary, YOLOv4-Tiny performed satisfactorily in detecting fish freshness, with a low rate of misclassification. By incorporating fluorescence imaging, the features of the samples are more obvious and the model has a good recognition performance. The main reasons affecting the fish freshness detection are environmental factors and model generalization ability. Due to the temperature difference between the experimental environment and the storage temperature, as well as the high humidity in summer, long-term exposure of samples may accelerate rot. The recommended approach is to minimize the exposure time of samples in the air during the experiment and conduct experiments in an environment where temperature and humidity can be better controlled. Since our training sample size is not big, when making predictions, the generalization performance on new samples may be not satisfactory. The recommended approach is to increase the number of training samples so that the model can learn more data features and patterns, thereby improving generalization performance.

### 4.3. Optimization Results of YOLOv4-Tiny Acceleration Circuit

To make full use of FPGA resources and speed up the freshness detection of grass carp fillet fluorescence images, the YOLOv4-Tiny circuit is optimized by using for-loop unrolling, for-loop pipeline, array partition, and ping-pong operation. Table 2 shows optimized and non-optimized time latency and resource utilization. Without optimization, the delay of the YOLOv4-Tiny circuit is 28.24 M cycles, and the circuit consumes 115 BRAM_18Ks, 93 DSPs, 20,046 FFs, and 21,161 LUTs, accounting for 41.07%, 42.27%, 18.84%, and 39.78% of the total FPGA resources, respectively. After optimization, the latency is 3.52 M cycles, which is only 12.46% of the non-optimized latency, and the resource consumption increases to 195 BRAM_18Ks, 213 DSPs, 47,102 FFs, and 41,020 LUTs, accounting for 69.64%, 96.82%, 44.27%, and 77.11% of the total FPGA resources, respectively. By contrast, the circuit with optimization consumes more resources in exchange for a significant reduction in latency, speeding up the reasoning of the detection algorithm while making full use of FPGA resources.

### 4.4. Evaluation of Other Performance of the Developed Device

For a fish freshness detecting device, power and detecting speed are also factors to be considered in addition to its detection accuracy. Therefore, the detection speed and power consumption of the developed device also was evaluated.

The detection time is mainly composed of the time of image acquisition and the subsequent processing time. The acquisition time of each sample is determined by the shutter setting time of the camera (set to 0.5 s in this study); the image processing time including the inference time of the detection model and the storage time of the resultant image is about 0.32 s. Therefore, the device can complete a sample test in less than 1 s, thus meeting the rapidity requirements of on-site inspection. The power consumption of the embedded main control board, including the LCD touch screen, CMOS camera and algorithmic circuits, is about 4.7 W as measured by a power meter; and the power consumption of the fluorescent excitation light source (including four 10 W UV-LED lamp beads and two 1.2 W cooling fans) is estimated to be about 42.4 W. Therefore, the power consumption of the whole device is about 47.1 W. Excluding the essential excitation light source power consumption (about 90% of the total consumption), the developed device meets the low-power requirements for embedded devices. In summary, the fluorescence imaging device developed in this study for fish freshness detection shows good advantages in power consumption and detection speed.

### 4.5. Comparison of the Developed Device with Other Devices

The device developed in this paper has been compared with other optical inspection devices for meat (fish) freshness detection in many aspects, including detection method, accuracy, portability and price. Table 3 shows the details of the performance of each device.

In Table 3, Chen et al. [36] used a hyperspectral device to classify the freshness of fish under different storage conditions by using the PLS-DA model with a detection accuracy of 97.62%. However, the device is not portable and the cost of the core components is high, and the overall price of the hyperspectral device is at least 400K RMB. Yakes et al. [37] introduced a handheld NIR spectrometer to analyze the freshness of red snapper and mahi-mahi fillets through the PLS-DA model, with an average accuracy of 78%. The device is easy to carry and the price is only 4–5K RMB. Zhuang et al. [22] established a fluorescence spectroscopy imaging system to detect pork freshness using the PLSR method, and the prediction accuracy of Rp was 0.94. The portable fluorescence technology studied has not yet been applied to the device, and the price of the whole system is in the range of 50K to 100K RMB. Wang et al. [38] used a portable Raman spectrometer to classify the freshness of fish through a CNN model, with a detection accuracy of 90.60%. The device is easy to carry, and the price of the device is 100–140K RMB.

Our device uses low-cost fluorescence imaging technology, and the overall price of the device is 1–2K RMB, which is an obvious price advantage among all the devices listed for meat (fish) freshness detection. The device integrates a YOLOv4-Tiny deep learning model to automatically extract image features, and fish freshness detection accuracy is 97.10%. Its accuracy is second only to the expensive high-resolution hyperspectral devices, but our device processes RGB image data, which is much smaller in terms of data volume and computational consumption compared to hyperspectral devices. At the same time, our device is portable and also has the advantages of low power consumption and high detection speed.

## 5. Conclusions

In this study, a portable rapid fish freshness detection device was developed based on fluorescence imaging technology using a low-cost FPGA board and a lightweight deep neural network (YOLOv4-Tiny model), and its performance (detection accuracy, detection time and power consumption), was evaluated by using grass carp fillets as the object. The power consumption of the developed device is about 47.1 W (including the power consumption of the excitation light source of 42.4 W and the power consumption of the MCU of 4.7 W), and the detection time for each sample is less than 1 s. Based on 141 test samples (fish fillets), it was demonstrated that the device can achieve an average detection accuracy of 97.1% for the three types of freshness grades.

It should be noted that physicochemical indicators (e.g., TVB-N, TVB-C, PH) reflecting the freshness grade are often of interest in some specific situations. How to optimize the developed device, including the hardware system, the prediction model, and the control and compensation of the operating environment, in order to meet the need for high-precision estimation of these physicochemical indexes will be a problem to be solved in the future.

## Figures and Tables

**Table 1:** Performance of YOLOv4-Tiny fluorescence image freshness detection model for grass carp fillets.

Class	Precision (%)	Recall (%)	F1-Score	AP (%)
Freshness	95.74	100.00	0.96	99.71
Secondary freshness	90.91	90.91	0.91	93.70
Rot	96.08	92.45	0.95	97.89

**Table 2:** Non-optimized and optimized latency and resource utilization.

	Latency (M Cycles)	BRAM _18Ks ^[a]^	DSPs	FFs ^[b]^	LUTs ^[c]^
Non-optimized	28.24	115 (41.07%)	93 (42.27%)	20,046 (18.84%)	21,161 (39.78%)
Optimized	3.52	195 (69.64%)	213 (96.82%)	47,102 (44.27%)	41,020 (77.11%)

Note: the total resources of the ZYNQ XC7Z020 development board include: 280 BRAMs, 220 DSPs, 106,400 FFs, and 53,200 LUTs. ^[a]^ BRAM_18K is dual-port 18 Kb BRAM. ^[b]^ FF (Flip Flop) is triggered along the clock and is the basic storage unit of the register. ^[c]^ LUT (look up table) is essentially a RAM.

**Table 3:** Evaluation of our device and other devices for detecting meat (fish) freshness.

Device	Method	Accuracy	Portability	Price (RMB) ^[a]^
Hyperspectral device [36]	HSI + PLS-DA ^[b]^	97.62%	No	400K+
NIR spectrometer [37]	NIR + PLS-DA ^[b]^	78%	Yes	4–5K
Fluorescence spectrometer [22]	Fluorescence spectroscopy + PLSR	TVB-N: Rp ^[c]^ = 0.94	No	50–100K
Raman spectrometer [38]	Raman spectroscopy + machine learning	90.60%	Yes	100–140K
Our device	Fluorescence imaging + YOLOv4-Tiny	97.10%	Yes	1–2K

^[a]^ The price of the device is obtained from the manufacturer’s website according to the model number of the device used. ^[b]^ PLS-DA is an abbreviation for partial least squares discriminant analysis. ^[c]^ Rp is an important indicator of the PLSR model that measures the predictive ability of the model. The closer its value is to 1, the better the predictive performance.

## Data Availability

Data are contained within the article.
